# Physiological and ecological characteristics of *Periploca sepium* Bunge under drought stress on shell sand in the Yellow River Delta of China

**DOI:** 10.1038/s41598-020-66717-z

**Published:** 2020-06-12

**Authors:** Xiao Wang, Jiang-Bao Xia, Xue-Bin Cao

**Affiliations:** 1 Shandong Key Laboratory of Eco-Environmental Science for the Yellow River Delta, Binzhou University, Binzhou, 256603 China; 2 National Algae and Sea Cucumber Project Technology Research Center, Shandong Oriental Ocean Sci-Tech Company Limited, Yantai, 264003 China

**Keywords:** Environmental impact, Forest ecology, Forestry

## Abstract

This study investigated the physiological and ecological changes in *P. sepium* Bunge and elucidated the physiological regulatory mechanisms underlying the adaptation of *P. sepium* to drought stress in shell sand. Drought stress led to a significant decrease in the net photosynthesis rate (*P*_n_) and respiration rate of leaves and a decrease in low-intensity light-use efficiency (*LUE*) and light ecological amplitude. An increase in drought stress led to a considerable decrease in the photosynthetic electron transport rate in the *P. sepium* leaves and a significant increase in the amount of light energy dissipated as heat. In addition, the photosynthesis process suffered from severe photoinhibition. *P. sepium* plants counteracted the effects of drought stress primarily by increasing their peroxidase (POD) activity and by regulating membrane lipid peroxidation by secreting greater numbers of osmotic adjustment substances (proline (Pro) and soluble sugars (Ss)) and malondialdehyde (MDA). As drought stress increased, both the stem sap flow rate and the cumulative sap flow of *P. sepium* decreased considerably. *P. sepium* Bunge adapts to drought stress through interregulatory activity between photosynthesis, water-related physiological activities, and physiological and biochemical processes, and this species exhibits relatively high adaptive plasticity to drought.

## Introduction

Shell ridges are unique beach ridge landforms near the high tide line that forms from the accumulation of residual shells from dead mollusks and their fragments, which are transported by waves. The shell ridges in the Yellow River Delta compose one of the three largest groups of ancient shell ridges in the world, and this group is the only one composed of both new and old shell ridges. Owing to its close proximity to a muddy coastal zone, the groundwater below these shell ridges is relatively shallow and is easily affected by natural factors, e.g., tides and high evaporation-precipitation ratios^1^. The soil in the beach ridge area of the shell ridges, which sits at a relatively high altitude, undergoes frequent dry/wet cycles but has been experiencing an increasing frequency of severe drought stress events. The soil in the beach ridge area of the shell ridges has a high porosity, a low capacity to store and retain water, and low capillary action. These properties have led to a lack of freshwater resources in the shell sand habitat. Soil water content is a key factor that limits plant growth and community succession in shell sand habitats^2^. *Periploca sepium* Bunge is a dominant shrub species on the shell ridges of the Yellow River Delta. Owing to its exceptional capability to disrupt wind, hold sand, resist drought, and retain water, *P. sepium* Bunge has become a primary tree species studied for its ability to persist under drought conditions^3,4^. During drought stress, the soil water content substantial affects plants in terms of their physiological, biochemical, and photosynthesis characteristics as well as their water-related physiological processes. Dominant plants can withstand stress by regulating their metabolic rhythm^2,5^. Therefore, studying the physiological and ecological characteristics of the dominant shrubs in shell sand on the Yellow River Delta under drought stress is of great importance for elucidating the mechanism through which xerophytic vegetation in the beach ridge area of shell ridges adapts to drought stress.

Plants adapt to stress conditions by continuously varying and adjusting their photosynthesis, water transport, physiological and biochemical characteristics. The light-compensation point (*LCP*), light-saturation point (*LSP*), and maximum net photosynthesis rate (*P*_max_) of plant leaves are the primary parameters used for measuring photosynthesis physiological processes in response to stress conditions^1,6^. Specifically, the initial fluorescence (*F*_0_), photochemical yield of photosystem II (*Φ*_PSII_), and nonphotochemical quenching coefficient (*NPQ*) are essential parameters for evaluating the photosynthesis regulatory mechanism of plants^7,8^. Enzyme activity is also a factor that can indicate changes in plant physiological activity^9,10^. Drought stress can cause increased activity of enzymes such as superoxide dismutase (SOD) and peroxidase (POD)^11^. Under suitable water conditions on shell ridges, *Tamarix chinensis* exhibited relatively high photosynthesis efficiency, although waterlogging and drought stress affected its photosynthesis efficiency by affecting leaf gas exchange^12^. Under mild drought stress, plants close there stomata. This action not only prevents the loss of water required for vital activities but also prevents atmospheric carbon dioxide (CO_2_) entering the leaves, resulting in a decrease in intercellular CO_2_ concentration; because of the deficiency in raw materials, photosynthesis is inhibited^13^. As drought stress increases, the photosynthesis organs in plants start to sustain damage. Additionally, the electron transport chain in photosystem II (PSII) can become damaged or even disrupted, resulting in a rapid decrease in photosynthesis efficiency and a sharp increase in the intercellular CO_2_ concentration. As a result, the stomatal function is also severely affected^13^. Therefore, the physiological regulatory mechanisms that plants use to adapt to drought stress can vary substantially depending on the soil texture, water conditions, and plant species.

In recent years, researchers have investigated the photosynthesis characteristics, water physiology, and physiological and biochemical processes of plants. However, most of these studies evaluated the effects of drought stress on individual physiological indices of xerophytes and focused primarily on the effects of drought stress on the photosynthesis parameters and physiological variations of typical xerophytic plants or crop species^14–17^. Vieira *et al*. reported that drought stress led to a decrease in the net photosynthesis rate (*P*_n_) and stomatal conductance (*G*_s_) of *Vateria macrocarpa* and an increase in its SOD, ascorbate peroxidase, and catalase activities. Additionally, the authors reported that these parameters recovered to control levels after rehydration^14^. Liu *et al*. reported that severe drought stress damaged or even disrupted the photosynthetic electron transport chain in maize, significantly decreasing the photosynthesis activity^15^. Similarly, Ou *et al*. reported a significant decrease in the *P*_max_ and dark respiration rate (*R*_d_) of *Excentrodendron hsienmu* and a significant increase in its osmotic adjustment substance and malondialdehyde (MDA) contents in a simulated arid karst habitat^16^. Xin *et al*. discovered that the stem sap flow rate and average daily stem sap flow of two *Populus* species from the Tibetan region are significantly lower during the dry season than during the rainy season and that the stem sap flow dynamics shift mostly from a multimodal pattern during the rainy season to a unimodal pattern during the dry season^17^. Plants suffer from photoinhibition caused by photosynthesis carbon assimilation and unbalanced water loss during drought stress^1,18^. Additionally, drought stress can also easily lead to the formation of reactive oxygen species and can disrupt the scavenging equilibrium in plants, resulting in membrane lipid peroxidation^19,20^. With respect to the relationships between the physiological and ecological characteristics of plants and water, there are generally two primary types of water treatment methods. One type focuses on water adaptability under mild, moderate, and severe stress. These methods address the patterns of plant adaptations to long-term drought stress by evaluating their physiological photosynthesis parameters and relevant mechanisms^21^. By using a serial, short-term, multilevel water gradient, the other method involves investigating the threshold effect of the soil water content on the physiological photosynthesis parameters of plants^1,5,12^. Physiological and ecological changes in plants under drought stress in shell sand habitats are less frequently studied. How changes in the water contents of shell sand affect the photosynthesis, water transport, and physiological and biochemical processes of the dominant species *P. sepium* Bunge along the shell ridges in the Yellow River Delta as well as the tolerance and adaptability of *P. sepium* Bunge to drought stress in shell sand remain unknown. Few studies have investigated the comprehensive photosynthesis efficiency, water physiology, and physiological and biochemical processes of *P. sepium* Bunge in shell sand habitats. The physiological and ecological regulatory mechanisms through which *P. sepium* Bunge adapts to drought stress in shell sand remain unclear. These factors somewhat hinder water management and the selection of habitats with suitable soil water contents, both of which are vital for guiding the planting of *P. sepium* Bunge in the degraded ecosystem of the shell ridges along the Yellow River Delta.

Hence, this study investigated four-year-old seedlings of *P. sepium* Bunge, a typical xerophytic shrub species that grows along the shell ridges in the Yellow River Delta, on four simulated shell sand habitats whose water conditions differ. Several *P. sepium* Bunge indices and parameters were measured, including leaf photosynthesis physiological indices and chlorophyll fluorescence parameters, stem sap flow parameters, and primary physiological and biochemical indices. The effects of drought stress on the physiological and ecological characteristics of *P. sepium* Bunge in shell sand habitats were evaluated. This study aimed to provide a reference for the water management of plant resources in dry shell sand habitats.

## Results

### Light responses according to the physiological photosynthesis indices

**Figure 1:**
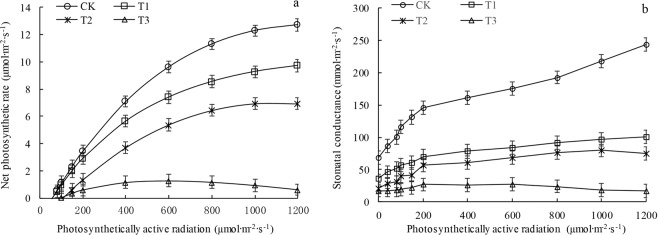
Light response of net photosynthesis rate (**a**) and stomatal conductance (**b**) of *P. sepium* leaves under various drought stress conditions.

As demonstrated in Fig. 1a, the rectangular hyperbolic correction model had a high goodness of fit with the *P*_n_–*PAR* response process in the leaves of *P. sepium* Bunge from the four shell sand habitats whose water conditions differed. The fitted correlation coefficients for the control (CK), T1, T2, and T3 groups were 0.998, 0.999, 0.995, and 0.932, respectively. As the drought stress increased, the *P*_n_ of *P. sepium* Bunge decreased significantly (*P* < 0.05). When the *PAR* was 1200 µmol∙m^−2^ ∙ s^−1^, the *P*_n_ values of *P. sepium* Bunge in the T1, T2, and T3 groups were 23.50%, 42.44%, and 95.12% lower than that in the CK, respectively. Figure 1b shows that as the drought stress increased, the *G*_s_ of the leaves decreased significantly (*P* < 0.05). When the *PAR* was 1200 µmol∙m^−2^ ∙ s^−1^, the *G*_s_ of the leaves in the CK was the greatest, at 243.56 mmol∙m^−2^∙s^−1^, and the *G*_s_ values in the T1, T2, and T3 groups were 41.42%, 30.88%, and 6.89% of that in the CK, respectively.

### Light-response parameters of photosynthesis

As the drought stress increased, the *LCP* of the leaves from *P. sepium* Bunge increased significantly (*P* < 0.05) (Table 1). The *LCP* values for the T1, T2, and T3 groups were 1.72%, 87.93%, and 67.24% greater than that for the CK, respectively. The *LSP* values of the leaves from the CK, T2, and T3 groups were 20.68%, 32.28%, and 63.64% lower than that from the T1 group, respectively. As the drought stress increased, both the apparent quantum yield (*AQY*) and *R*_d_ of the leaves decreased significantly (*P* < 0.05). The leaf *P*_max_ was the greatest in the CK, followed by the T1, T2, and T3 groups. The *P*_max_ values of the leaves in the T1, T2, and T3 groups were 78.68%, 54.86%, and 9.95% of the value in the CK, respectively.

**Table 1:** Light-compensation point (*LCP*), light-saturation point (*LSP*), apparent quantum yield (*AQY*), dark respiration rate (*R*_d_) and light-saturated net photosynthesis rate (*P*_max_) of *P. sepium* leaves under various drought stress conditions.

Water treatment	*LSP* (µmol∙m^−2^ ∙ s^−2^)	*LCP* (µmol∙m^−2^ ∙ s^−2^)	*AQY* (mol·mol^−1^)	*R*_d_ (µmol∙m^−2^ ∙ s^−2^)	*P*_max_ (µmol∙m^−2^ ∙ s^−2^)
CK	1285 ± 130^b^	58 ± 4^c^	0.03 ± 0.0015^a^	1.64 ± 0.07^a^	12.76 ± 1.21^a^
T1	1620 ± 113^a^	59 ± 5^c^	0.029 ± 0.0014^a^	1.57 ± 0.06^a^	10.04 ± 0.54^b^
T2	1097 ± 85^c^	109 ± 4^a^	0.018 ± 0.0019^b^	1.28 ± 0.15^b^	7.12 ± 0.96^c^
T3	589 ± 137^d^	97 ± 3^b^	0.012 ± 0.0021^c^	0.95 ± 0.12^c^	1.27 ± 0.57^d^

Note: The different lowercase letters indicate a significant difference between the groups under various water conditions (*P* < 0.05); the identical letters indicate no significant difference (*P* > 0.05). The same scheme applies below.

### Chlorophyll fluorescence characteristics

**Figure 2:**
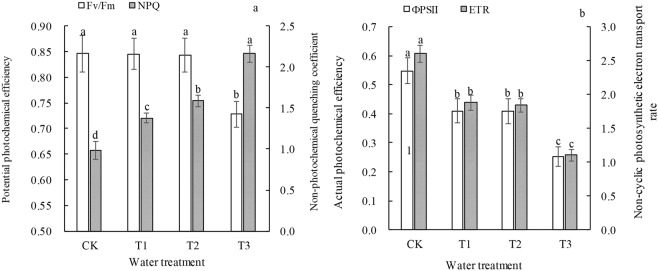
Potential photochemical efficiency and nonphotochemical quenching coefficient (**a**) as well as the actual photochemical efficiency and noncyclic photosynthetic electron transport rate (**b**) of leaves of *P. sepium* under various drought stress conditions.

As the drought stress level increased, the *F*_v_*/F*_m_ ratio of the *P. sepium* Bunge leaves decreased significantly (*P* < 0.05). However, there was no significant difference in the *F*_v_*/F*_m_ ratio between the CK, T1, and T2 groups (*P* > 0.05) (Fig. 2). The *NPQ* values of the leaves in the T1, T2, and T3 groups were 40.10%, 61.44%, and 119.88% greater than that in the CK, respectively. The effects of drought stress on the *ETR* and *Φ*_PSII_ of the leaves exhibited the same pattern. As the drought stress increased, there was a significant decrease in the leaf *ETR* (*P* < 0.05). The *ETR*s in the leaves in the T1, T2, and T3 groups were 27.84%, 32.14%, and 57.65% lower than that in the CK, respectively.

### Physiological and biochemical characteristics

**Figure 3:**
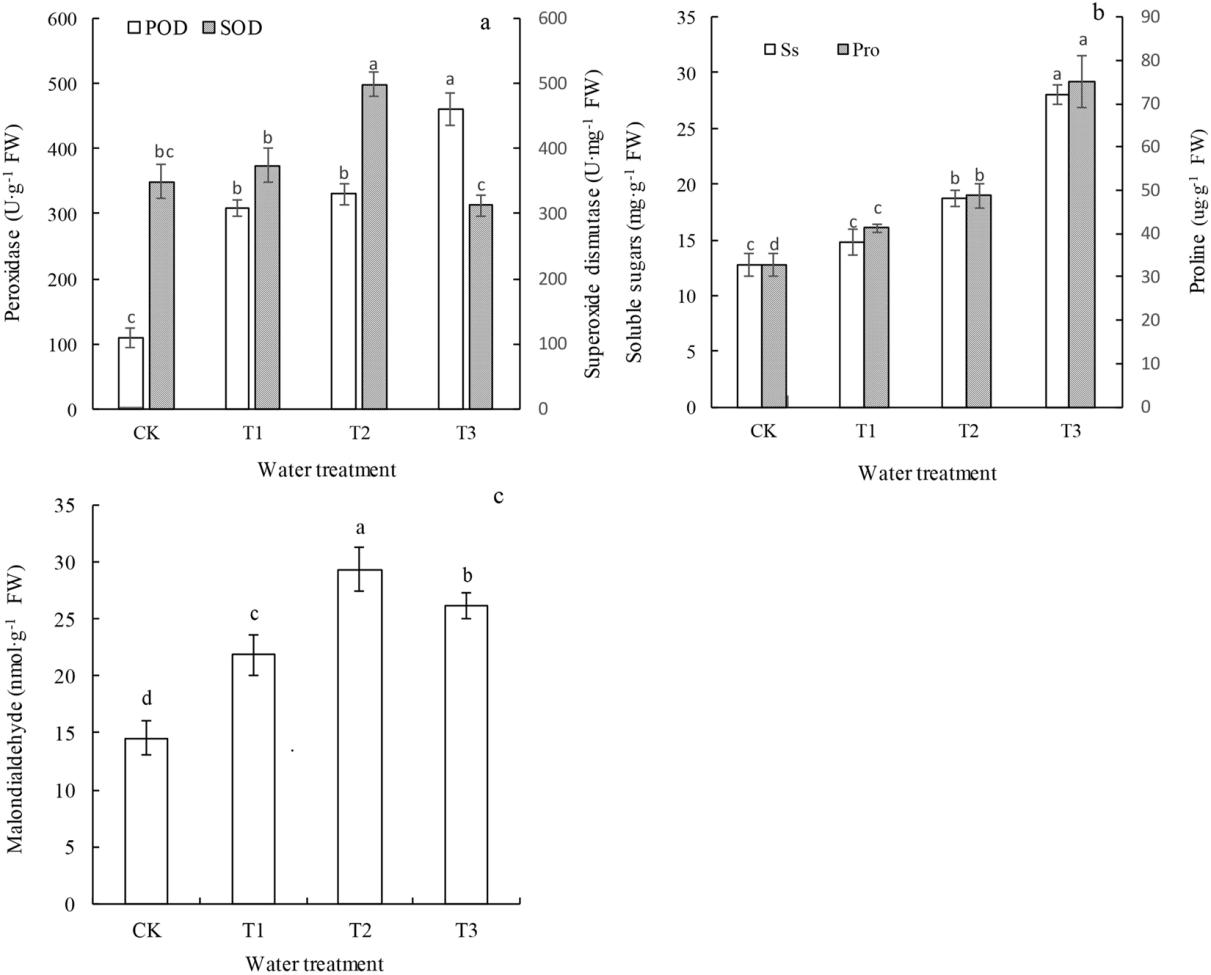
Superoxide dismutase and peroxidase activity (**a**), proline and soluble sugar contents (**b**) and malondialdehyde content (**c**) of leaves of *P. sepium* under various drought stress conditions.

As the drought stress increased, the SOD activity first increased but then decreased, and the POD activity increased (Fig. 3a). The POD activity in the T1, T2, and T3 groups was 2.80, 3.01, and 4.19 times that in the CK, respectively. As the drought stress increased, there was a significant increase in both the proline (Pro) and soluble sugar (Ss) (two types of osmotic adjustment substances) contents in the leaves (*P* < 0.05) (Fig. 3b). The MDA contents in the T1, T2, and T3 groups were 1.50, 2.02, and 1.80 times that in the CK, respectively (Fig. 3c).

### Changes in stem sap flow

**Figure 4:**
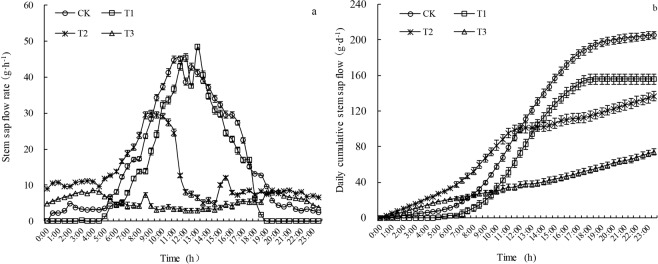
Daily stem sap flow rate dynamics (**a**) and daily cumulative stem sap flow (**b**) in *P. sepium* under various drought stress conditions.

There was a significant difference in the daily stem sap flow rate dynamics in *P. sepium* Bunge under the different water treatments (*P* < 0.05) (Fig. 4a). Under mild drought stress, the stem sap flow activity in *P. sepium* Bunge occurred primarily between 4:30 and 19:00. Under the other water conditions, stem sap flow activity occurred throughout the day. As the drought stress increased, the stem sap flow rate decreased overall. The daily cumulative stem sap flow was the greatest in the CK, followed by the T1, T2, and T3 groups (Fig. 4b). The daily cumulative stem sap flow rates in the T1, T2, and T3 groups were 23.98%, 33.3%, and 63.94% lower than that (205.21 g·d^−1^) in the CK, respectively. The daily cumulative stem sap flow in the T3 group increased slowly and linearly. The slope of the daily cumulative stem sap flow curve for the T2 group decreased over time, and the curve exhibited an inverted “Z” shape; the daily cumulative stem sap flow exhibited a typical “S”-shaped pattern in the CK and T1 groups.

### Changes in the *T*_r_ and *WUE*

**Figure 5:**
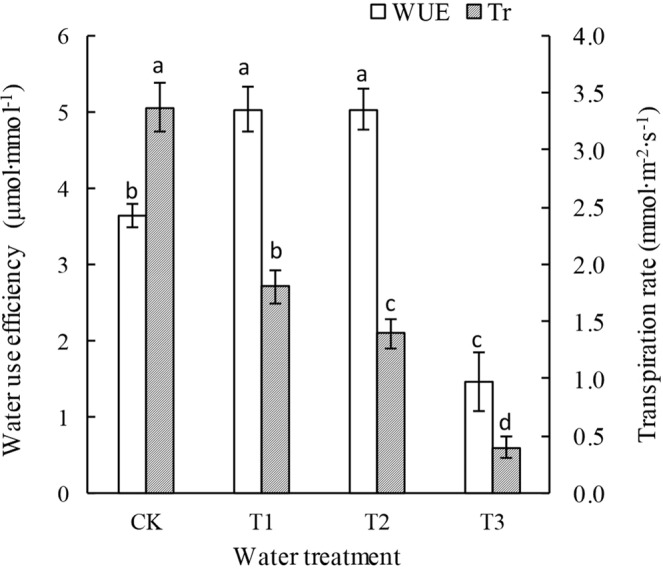
Transpiration rate and water-use efficiency of leaves of *P. sepium* under various drought stress conditions.

There was a significant difference in the *T*_r_ values of the *P. sepium* Bunge leaves under different drought stress levels (*P* < 0.05). The *T*_r_ was the greatest in the CK, followed by the T1, T2, and T3 groups (Fig. 5). When the *PAR* was 1200 µmol∙m^−2^∙s^−1^, the *T*_r_ results for the T1, T2, and T3 groups were 50.82%, 37.16%, and 10.11% of that (3.66 mmol∙m^−2^∙s^−1^) in the CK. As the drought stress increased, the water-use efficiency (*WUE*) of the leaves first increased but then decreased significantly (*P* < 0.05). The *WUE* remained at a relatively high level in the T1 and T2 groups but was the lowest in the T3 group. When the *PAR* was 1200 µmol∙m^−2^∙s^−1^, the *WUE* values of the leaves in the T1 and T2 groups were 37.87% and 38.09% greater than that in the CK, respectively, whereas the *WUE* of the leaves in the T3 group was 59.84% lower than that in the CK.

## Discussion

There was a significant difference in the extent of the decreases in the *P*_n_ and *G*_s_ between the *P. sepium* leaves in the shell sand habitats under different drought stress conditions. The decrease in the photosynthesis efficiency of the leaves is related to their stomatal state^12^. Research has shown a significant decrease in both the *P*_n_ and *G*_s_ of *Populus* × *euramericana* ‘Neva’ and *Phaseolus vulgaris* L., a difference in the stomatal limitation mechanism between these two species, and a significant positive correlation between the *P*_n_ and *G*_s_ under various water stress conditions^22,23^. These findings are similar to those of the present study. The leaves of *P. sepium* Bunge adapt to drought stress in shell sand via stomatal regulation. This mechanism varies significantly with the drought stress level. As the drought stress level increased, the *P*_n_ of the leaves of the four-year-old *P. sepium* Bunge seedlings decreased significantly, whereas the *P*_n_ of the leaves of one-year-old *P. sepium* Bunge seedlings first increased but then decreased^24^. This may be because young *P. sepium* Bunge seedlings are more sensitive to drought stress. The response of the low-intensity light-use efficiency (*LUE*) of the leaves to drought stress is nonsignificant. However, the low-intensity *LUE* of the leaves can decrease significantly under severe drought stress. Under natural conditions, the *AQY* of plant leaves ranges from 0.03 to 0.05 mol·mol^−1^^25^. In the present study, the *AQY* of the leaves from the shell sand habitats was significantly lower than this range. This result suggests that the photosynthesis capability of *P. sepium* Bunge is relatively weak under low-intensity light. The difference between the *LSP* and *LCP* of the leaves under severe drought stress was only 40.10% of that in the CK. This finding indicates that drought stress can significantly reduce the *LUE* and light ecological amplitude of leaves during photosynthesis. Consistent changes in the *R*_d_ and *AOY* of the *P. sepium* Bunge leaves under drought stress were detected. This finding suggests that leaves can adapt to drought stress by decreasing respiration. Waterlogging and drought stress resulted in a significant decrease in the *AQY*, *P*_max_, and *LSP* of leaves of *T. chinensis* growing in a shell sand habitat, and there was a significant increase in their *LCP*^12^. These findings are similar to those of the present study. The *AQY* and *R*_d_ of *Aralia elata* first increased but then decreased as drought stress increased, and both of its *AQY-*drought stress and *R*_d_*-*drought stress curves exhibited an “n” shape^26^. While the *AQY* and *R*_d_ of *A. elata*^26^ start to decrease at a lower water stress level than did those of *P. sepium* Bunge on shell sand ridges, the *AQY* and *R*_d_ of *A. elata* decrease more sharply as the water stress level increases. Thus, *P. sepium* Bunge exhibits a greater *LUE* under drought stress than under no drought stress.

A significant decrease in the *F*_v_*/F*_m_ ratios of leaves of *P. sepium* Bunge growing in the shell sand habitat under severe drought stress was detected. This finding suggests that under these conditions, there was a decrease in the photochemical quantum yield of PSII in the electron transport chain in the leaves, and the photosynthesis process suffered from severe photoinhibition^21,27^. Under severe drought stress, the leaves suffered from severe photoinhibition, and there was a significant decrease in the number of electrons for the light reactions or CO_2_ assimilation and a decrease in photosynthesis efficiency^21,28^. A significant positive correlation was detected between the changes in the *ETR* and *Φ*_PSII_^28^. Under mild drought stress (relative water content (*RWC*) > 40%), there was a relatively insignificant decrease in the *NPQ* of the leaves, which had a relatively low heat dissipation capacity. Drought stress at this level did not cause damage to the photosynthesis organs of the leaves. Under mild drought stress, the leaf photosynthesis activity was relatively insignificantly inhibited and could be restored by measures such as rehydration^12,29^. Under severe drought stress (*RWC* ≤ 40%), there was a significant decrease in the *NPQ* of the leaves and a significant increase in their heat dissipation. Additionally, under severe drought stress, the photosynthesis ability of the leaves could be easily inhibited and could not be restored^12,29^. The *P*_n_ of the leaves was extremely and significantly negatively correlated with the *NPQ* values but significantly positively correlated with *Ф*_PSII_, *ETR*, and *R*_d_ (Table 2). This finding suggests that orderly electron transport along the electron transport chain is the determinant for photosynthesis output and that light energy dissipation is a key protective mechanism of photosynthesis under stress conditions^27,28^. In addition, respiration also regulates normal photosynthesis output to a certain extent. A decrease in the soil water content led to a significant decrease in the *F*_v_*/F*_m_ ratio, *Φ*_PSII_, and *ETR* of four wolfberry species and a significant increase in their *NPQ*^30^. However, compared with that of *P. sepium* Bunge, the decrease in the *F*_v_*/F*_m_ ratio of these four wolfberry species was more pronounced. Silva *et al*. reported that, under various water stress conditions, there was no significant difference in the *F*_v_*/F*_m_ ratio of *Jatropha curcas*, a significant decrease in its *ETR*, and a significant increase in its *NPQ*^31^. Cecilia *et al*.^32^ reported that, increasing in excessive electron transport and non-photochemical quenching was shown on *Celtis australis* due to photosynthesis and gas exchange processes inhibited, and *C. australis* showed conserved water utilization behavior and was sensitive to drought stress^32^. These changes are consistent with those observed in this study. The effects of drought stress on the physiological photosynthesis processes of various plant species across various habitats can evidently differ relatively significantly.

**Table 2:** Correlation coefficients of photosynthesis and physiological water indices of *P. sepium*.

Index	*P*_n_	*T*_r_	*G*_s_	*WUE*	*F*_v_/*F*_m_	*Ф*_PSII_	*ETR*	*NPQ*	POD	SOD	MDA	Ss	Pro	*AQY*	*LSP*	*P*_max_	*LCP*	*R*_d_	CSF
*P*_n_	1	0.948	0.913	0.660	0.897	0.969^*^	0.968^*^	−0.997^**^	−0.940	0.181	−0.725	−0.990^*^	−0.994^**^	0.946	0.838	0.999^**^	−0.722	0.982^*^	0.993^**^
*T*_r_		1	0.995^**^	0.404	0.734	0.975^*^	0.984^*^	−0.970^*^	−0.998^**^	−0.007	−0.854	−0.894	−0.912	0.873	0.638	0.944	−0.722	0.898	0.978^*^
*G*_s_			1	0.324	0.674	0.959^*^	0.971^*^	−0.943	−0.996^**^	−0.049	−0.867	−0.847	−0.870	0.829	0.562	0.908	−0.696	0.852	0.954^*^
*WUE*				1	0.917	0.566	0.534	−0.607	−0.406	0.737	0.012	−0.751	−0.738	0.585	0.829	0.652	−0.201	0.676	0.583
*F*_v_/*F*_m_					1	0.848	0.827	−0.869	−0.737	0.568	−0.347	−0.939	−0.941	0.796	0.873	0.888	−0.434	0.879	0.855
*Ф*_PSII_						1	0.999^**^	−0.983^*^	−0.981^*^	0.217	−0.717	−0.935	−0.957^*^	0.849	0.686	0.959^*^	−0.602	0.906	0.987^*^
*ETR*							1	−0.984^*^	−0.989^*^	0.173	−0.747	−0.928	−0.950^*^	0.855	0.675	0.959^*^	−0.626	0.906	0.989^*^
*NPQ*								1	0.965^*^	−0.150	0.756	0.975^*^	0.984^*^	−0.931	−0.792	−0.994^**^	0.717	−0.967^*^	−0.999^**^
POD									1	−0.038	0.826	0.883	0.906	−0.843	−0.607	−0.933	0.672	−0.877	−0.974^*^
SOD										1	0.524	−0.257	−0.281	−0.045	0.235	0.149	0.488	0.107	0.136
MDA											1	0.644	0.644	−0.790	−0.457	−0.740	0.892	−0.728	−0.768
Ss												1	0.997^**^	−0.947	−0.900	−0.990^**^	0.698	−0.987^*^	−0.967^*^
Pro													1	−0.928	−0.866	−0.991^**^	0.665	−0.976^*^	−0.977^*^
*AQY*														1	0.903	0.959^*^	−0.890	0.986^*^	0.922
*LSP*															1	0.852	−0.707	0.912	0.770
*P*_max_																1	−0.750	0.989^*^	0.990^*^
*LCP*																	1	−0.804	−0.712
*R*_d_																		1	0.959^*^
CSF																			1

* means a significant correlation at the 0.05 level (two-tailed); ** means a significant correlation at the 0.01 level (two-tailed);

CSF, cumulative sap flow.

Under moderate drought stress, the SOD activity in the *P. sepium* Bunge leaves was relatively highly adaptable and adjustable. However, as the drought stress level increased, the ability to alter the SOD activity in the leaves was inhibited. As a result, there was a decrease in SOD activity. In comparison, there was a significant increase in POD activity. *P. sepium* Bunge evidently eliminates drought stress-induced peroxide stress primarily by increasing POD activity^31,33^. Studies have indicated that there is a significant increase in SOD, POD, and catalase activities in the leaves of *Haloxylon ammodendron*^34^ and peanut seedlings^35^ under various drought conditions. This finding suggests that, under drought stress, changes in the activities of plant protective enzymes are closely related to the habitat and drought resistance of the plants. As drought stress increases, the activities of protective antioxidant enzymes in plants increase or first increase but then decrease^33,36^. However, under severe drought stress, the ability of plants to alter their enzyme activities is limited, and consequently, their enzyme activities mostly decrease^33,36^.

Lü *et al*. reported a significant increase in the Pro and Ss contents in *H. ammodendron* under various drought stress conditions, although the Ss content increased to a greater extent than did the Pro content^34^. Similarly, Schimpl *et al*. reported a significant increase in the Ss content of *Bertholletia excelsa* under drought stress^37^. In the present study, as the drought stress increased, the Pro content in the leaves increased to a significantly greater extent than did the Pro content. This finding suggests that *P. sepium* Bunge is more capable of improving its osmotic adjustability by altering the Pro content than the Ss content. Under drought stress, plants can reduce their osmotic potential by accumulating solutes such as Pro and Ss to improve the osmotic adjustability of their cells to ensure proper osmotic balance inside and outside their cells^37–39^. In the present study, in the leaves, the Pro content was extremely significantly negatively correlated with the *P*_n_; significantly negatively correlated with the *ETR*, *Ф*_PSII_, and *R*_d_; and significantly positively correlated with the *NPQ*. Additionally, in the leaves, the Ss content was significantly negatively correlated with the *P*_n_ and *R*_d_; significantly positively correlated with the *NPQ*; and extremely significantly positively correlated with the Pro and Ss contents (Table 2). These findings suggest that, by secreting osmotic adjustment substances, *P. sepium* Bunge can adjust the osmotic pressure on both sides of the plasma membrane, and ensuring orderly physiological activity^37,38^, thereby affecting its photosynthetic electron transport ability. In the present study, as the drought stress increased, there was an increase in the contents of osmotic adjustment substances (e.g., Pro and Ss) in the *P. sepium* Bunge leaves as well as an increase in the activities of their antioxidant enzymes (SOD and POD). This trend may be the cause of the decrease in the MDA content^40,41^, which led to a reduction in cell membrane lipid peroxidation^36^. Abida *et al*.^42^ reported that two different varieties of maize resisted effectively drought stress by increasing antioxidant enzyme activity, reducing cell membrane lipid peroxidation efficiency and accumulating osmotic adjustive substances. As a result, the toxic effects of cell membrane lipid peroxidation diminished under severe drought stress.

As the drought stress level increased, the time at which the stem sap flow in *P. sepium* Bunge peaked was delayed or advanced, and the sap flow activity diminished during the day and increased during the night. To adapt to drought stress, *P. sepium* Bunge improved its *WUE* by reducing its transpiration intensity and duration. Furthermore, *P. sepium* Bunge exhibited relatively high adaptive plasticity to the dry soil environment. Bhusal *et al*. reported a significant decrease in instantaneous sap flow rate under drought stress^43^. Similarly, Liu *et al*. reported, that under drought stress, there was a decrease in the instantaneous sap flow rate of several dominant species in a *Quercus liaotungensis* community in a hilly loess region, a significant decrease in their maximum sap flow rates, and an advance or delay in the time at which the sap flow process started^44^. Clearly, drought stress obstructs the water transport process in plants and reduces their usable water content. In the present study, the *P*_n_ of the *P. sepium* Bunge leaves was extremely significantly positively correlated with the cumulative sap flow; the *T*_r_ was extremely significantly positively correlated with *G*_s_, significantly positively correlated with the cumulative sap flow, and significantly negatively correlated with the *NPQ*; and *G*_s_ was significantly positively correlated with the *Ф*_PSII_, *ETR*, and cumulative sap flow (Table 2). These results suggest that transpiration is closely related to the state of stomata. The photosynthetic electron transport and light energy dissipation processes also affected the *T*_r_ and *G*_s_ while protecting the photosynthesis organs, thereby intervening in the water-related physiological processes of *P. sepium* Bunge. Mengesha *et al*.^45^ reported that decreasing net photosynthetic rate and being limited gas exchange were shown on two types of wheat with different genotypes, which result in declining water transport and evapotranspiration rate, but improving significantly water use efficiency. Water transport and use in plants also affects their normal photosynthesis output. Certain relationships evidently exist between photosynthesis and water-related physiological processes in plants.

Under mild and moderate drought stress (*RWC* > 40%), there was a significant decrease in the *T*_r_ and *P*_n_ of the leaves (*P* < 0.05) (the *P*_n_ decreased to a smaller extent than did the *T*_r_) and an increase in their *WUE*. As the drought stress increased (*RWC* ≤ 40%), the *T*_r_ and *P*_n_ decreased to extremely low levels and were not significantly different (*P* > 0.05). Additionally, as the drought stress increased (*RWC* ≤ 40%), there was also a significant decrease in *WUE* (*P* < 0.05). Therefore, increasing its peroxidase activity and secreting more osmotic adjustment, the leaves of *P. sepium* Bunge exhibited a relatively high *WUE*. A decrease in *G*_s_ reduces the water consumption of plants due to transpiration, obstructs water transport and use processes, and inhibits water-related physiological activities. A decrease in *WUE* leads to a decrease in the amount of raw materials for photosynthesis, thereby reducing photosynthesis. Liang *et al*. reported a significant decrease in the *T*_r_, *P*_n_, and *WUE* of *Populus* × *euramericana* ‘Neva’ as the drought stress level increased^22^. Similarly, Xia *et al*. reported that, as the drought stress level increased, the *T*_r_ and *P*_n_ of *T. chinensis* in a shell sand habitat decreased, whereas its *WUE* first increased but then decreased^46^. These findings are consistent with those of this study. Drought stress results in a significant decrease in the *WUE* and transpiration-related water consumption of plants, thereby inhibiting their water-related physiological processes. This trend ensures that plants will be able to use limited water efficiently to survive drought stress.

## Conclusions

Drought stress on shell sand can significantly affect the photosynthesis, water consumption, and physiological and biochemical processes of *P. sepium* Bunge. As the degree of drought in shell sand area increased, a significant decrease in the *P*_n_, *T*_r_, *G*_s_, and light energy for photosynthesis of *P. sepium* Bunge leaves occurred, and an increased amount of light energy was dissipated as heat. Moderate drought stress significantly improved the *WUE*, and severe drought stress led to severe photoinhibition and obstructed the water transport of *P. sepium* Bunge. Drought stress leads to a decrease in the amount of water available for other physiological activities. The decrease in photosynthesis is an important photoprotection mechanism that enables *P. sepium* Bunge to adapt to drought stress on shell sand. Moreover, *P. sepium* Bunge counteracts lipid peroxidation by increasing the contents of osmotic adjustment substances (Pro and Ss) and activities of antioxidant enzymes (SOD and POD) under severe drought stress on shell sand and adapts to drought stress through interactions and interregulatory activity between photosynthesis, water-related physiological activities, and physiological and biochemical processes. By reducing its photosynthesis output and *WUE*, *P. sepium* Bunge can reduce its photosynthetic electron transport efficiency, increase heat dissipation, increase both the activities of its protective enzymes and the content of its lipid peroxide molecules, and improve the osmotic adjustability of its cell membranes to withstand and adapt to drought stress on shell sand.

## Methods

### Experimental materials

Four-year-old seedlings of *P. sepium* Bunge, a dominant shrub species that grows on the shell ridges of the Yellow River Delta, were selected as experimental materials. The *P. sepium* Bunge seedlings were uniformly cut to prepare samples that had a height of 1.18 ± 0.13 m, a rhizome thickness of 1.25 ± 0.06 cm and a root depth of 11.2 ± 0.3 cm. The seedling samples were subsequently trimmed such that the canopy size was 0.4 m (east–west) × 0.4 m (south–north).

### Experimental design

This experiment was conducted in the Scientific Research Greenhouse (SRG) of the Shandong Key Laboratory of Eco-Environmental Science for the Yellow River Delta. Shell sand was collected from the area of the *P. sepium* Bunge community in the Binzhou National Shell Ridge and Wetland Nature Reserve. The collected shell sand was sieved through a 2.0 mm mesh and was subsequently transferred to a pot (diameter of the top, 40 cm; height, 50 cm). The basic chemical and physical properties of the shell sand were as follows: field capacity, 18.31%; bulk density, 1.29 g·cm^−3^; particle size, 0.2–2.0 mm; pH, 7.40; and salt content, 0.1%–0.4%. The primary environmental conditions of the SRG included the following: illumination intensity, 80%–84% of the natural external light intensity relative air humidity, 45%–65%; temperature, 20–35 °C; and atmospheric CO_2_ concentration, 350–370 μmol·mol^−1^.

The seedlings were planted in a total of 24 pots (three seedlings per pot) in the SRG on March 13, 2018, after which they were subjected to normal plant management practices for approximately 90 d. On June 15, 2018, the seedlings received one of four water treatments. The seedlings were randomly divided into four groups, with each group comprising six pots of seedlings. One of the four groups was selected as a CK (*RWC* of the shell sand: 77.72%). The remaining three groups were subjected to mild (*RWC* of the shell sand: 58.16%), moderate (*RWC* of the shell sand: 42.98%), or severe (*RWC* of the shell sand: 32.39%) drought stress treatments, which are referred to as the T1, T2, and T3 groups, respectively. The pots in the CK, T1, and T2 groups were watered once every 3 days with 4, 2, and 1 L of water, respectively. The pots in the T3 group were not watered. The *RWC* of the shell sand was calculated as the ratio of the water content of the shell sand to its field capacity, by weight. The *RWC* of the shell sand was monitored and controlled by weighing the pot and drying. A tray was placed underneath each pot to prevent water loss, thereby controlling the designed water treatment and ensuring experimental accuracy. Each group of seedlings was allowed to adapt to the water treatment for approximately 30 d. Afterward, from July 16 to July 23, 2018, the *P. sepium* Bunge seedlings were measured to determine the physiological photosynthesis indices and the chlorophyll fluorescence parameters of their leaves as well as their physiological and biochemical indices and stem sap flow parameters.

### Measurement of physiological photosynthesis indices

The experiment was conducted from 9:00 to 11:30 on a sunny day. Three pots of *P. sepium* Bunge seedlings were randomly selected from each group as experimental samples. Three healthy, mature leaves were then selected from the middle-upper section of the seedlings in each pot. An LI-6400XT portable photosynthesis system (LI-COR, Inc., Lincoln, NE, USA) was used to measure the light response of the plants. A standard 6400-02B red and blue light-source leaf chamber was used. Photosynthetically active radiation (*PAR*) was subsequently applied at 0, 40, 80, 100, 150, 200, 400, 600, 800, 1000, and 1200 µmol∙m^−2^ ∙ s^−1^, for a total of 11 illumination intensities. During the measurements, the *PAR* was decreased from the greatest level to the lowest level, with each level maintained for 120 s; these measurements were repeated three times. The instrument automatically recorded and calculated various physiological photosynthesis indices, such as the *P*_n_, transpiration rate (*T*_r_), *G*_s_, and *WUE* (*WUE* = *P*_n_/*T*_r_).

### Measurements of chlorophyll fluorescence parameters

An FMS-2 portable pulse-modulated fluorometer (Hansatech Instruments, Ltd., UK) was used to measure the chlorophyll fluorescence parameters of the *P. sepium* Bunge leaf samples as well as their photosynthesis indices. After the leaves were dark adapted for 30 min, the *F*_0_ and the maximum fluorescence (*F*_m_) of each leaf were measured. Subsequently, after each leaf was dark adapted to natural light for 50 min, it was measured to determine its steady-state and maximum fluorescence under light-adapted conditions (*F*_s_ and *F*_m_′). Additionally, the potential photochemical efficiency (*F*_v_/*F*_m_ = (*F*_m_ − *F*_0_)/*F*_m_), actual photochemical efficiency (*Φ*_PSII_ = (*F*_m_′ − *F*_s_)/*F*_m_′), noncyclic photosynthetic electron transport rate (*ETR* = *Φ*_PSII_ × *PAR* × 0.5 × 0.84, where 0.5 is the partition coefficient between the two photosystems and 0.84 is the proportion of the amount of light absorbed by the leaf to the amount of incident light), and *NPQ* ((*F*_m_ − *F*_m_′)/*F*_m_′) were calculated^47^.

### Measurements of physiological and biochemical indices

To measure physiological and biochemical indices, five to seven leaves were collected from the same section of each seedling from which the leaves were collected to measure and analyze the photosynthesis parameters^48^. The SOD activity was measured via nitro blue tetrazolium photoreduction, and the POD activity was measured via guaiacol colorimetry. In addition, the Pro content was determined via ninhydrin colorimetry, and the Ss content was determined according to the anthrone coloration method. Last, the MDA content was measured via thiobarbituric acid colorimetry.

### Measurements of stem sap flow parameters

A Flow 32 heat-balance stem sap flow measurement system (Dynamax, Houston, TX, USA) was used to measure the *P. sepium* Bunge seedling stem sap flow rate and daily stem sap flow continuously. On the basis of the stem thickness for each *P. sepium* Bunge seedling selected for testing, an SGA-5 sensor with a suitable diameter (5–7 mm) was selected. The stem sap flow measurement system was installed according to the standard instructions provided by Dynamax. A Delta-T Logger data acquisition system was used to collect and record the instantaneous stem sap flow rates automatically at 30 min intervals.

### Statistical analysis

A rectangular hyperbolic correction model^49^ was used to simulate the *PAR*–*P*_n_ photosynthesis light-response process. Equation (1) shows the expression of the model as follows:

1 $${P}_{n}=\alpha \frac{1-\beta I}{1+\gamma I}(I-{I}_{c})$$

Here, *P*_n_ is the net photosynthesis rate; *I* is the *PAR*; *I*_c_ is the *LCP*; and *α*, *β*, and *γ* are three coefficients unrelated to the light intensity (*α* is the initial slope of the light-response curve when the *PAR* is 0, which is considered the *AQY*, and *β* and *γ* are biological important photoinhibition and photosaturation terms, respectively).

On the basis of Eq. (1), three photosynthesis parameters, namely, *R*_d_, *LSP*, and *P*_max_, can be derived, as shown in Eqs. (2)–(4).

2 $${{R}}_{{d}}={P}({I}=0)=-\,\alpha {{I}}_{{c}}$$

3 $${I}_{m}=\frac{-1+\sqrt{\frac{(\beta +\gamma )(1+\gamma {I}_{c})}{\beta }}}{\gamma }$$

where *I*_m_ is the *LSP*.

4 $${P}_{n}({I}_{m})=\alpha \frac{1-\beta {I}_{m}}{1+\gamma {I}_{m}}({I}_{m}-{I}_{C})$$

where *P*_n_(*I*_m_) is equal to *P*_max_.

The data were processed and plotted in Microsoft Excel 2016. Correlation analysis, analysis of variance, and multiple comparisons of the data were performed by SPSS 19.0.
